# A corner reflector of graphene Dirac fermions as a phonon-scattering sensor

**DOI:** 10.1038/s41467-019-10326-6

**Published:** 2019-06-03

**Authors:** H. Graef, Q. Wilmart, M. Rosticher, D. Mele, L. Banszerus, C. Stampfer, T. Taniguchi, K. Watanabe, J.-M. Berroir, E. Bocquillon, G. Fève, E. H. T. Teo, B. Plaçais

**Affiliations:** 1 Laboratoire de Physique de l’Ecole normale supérieure, ENS, Université PSL, CNRS, Sorbonne Université, Université Paris-Diderot, Sorbonne Paris Cité, Paris, 75005 France; 2 CINTRA, UMI 3288, CNRS/NTU/Thales, Research Techno Plaza, 50 Nanyang Drive, Singapore, 637553 Singapore; 3 School of Electrical and Electronic Engineering, Nanyang Technological University, 50 Nanyang Avenue, Singapore, 639798 Singapore; 4 2nd Institute of Physics, RWTH Aachen University, 52074 Aachen, Germany; 5 Advanced Materials Laboratory, National Institute for Materials Science, Tsukuba, 305-0047 Ibaraki Japan; 6 School of Materials Science and Engineering, Nanyang Technological University, 50 Nanyang Avenue, Singapore, 639798 Singapore

**Keywords:** Nanoscience and technology, Physics

## Abstract

Dirac fermion optics exploits the refraction of chiral fermions across optics-inspired Klein-tunneling barriers defined by high-transparency p-n junctions. We consider the corner reflector (CR) geometry introduced in optics or radars. We fabricate Dirac fermion CRs using bottom-gate-defined barriers in hBN-encapsulated graphene. By suppressing transmission upon multiple internal reflections, CRs are sensitive to minute phonon scattering rates. Here we report on doping-independent CR transmission in quantitative agreement with a simple scattering model including thermal phonon scattering. As a signature of CRs, we observe Fabry-Pérot oscillations at low temperature, consistent with single-path reflections. Finally, we demonstrate high-frequency operation which promotes CRs as fast phonon detectors. Our work establishes the relevance of Dirac fermion optics in graphene and opens a route for its implementation in topological Dirac matter.

## Abstract (web summary)

Dirac fermion optics leverages p-n junctions and Klein tunnelling barriers present in materials to implement complex optical functions and devices, including reflectors, collimators, and Dirac fermion microscopes. Here, the authors fabricate Dirac fermion corner reflectors using bottom-gate-defined barriers in hBN-encapsulated graphene, and demonstrate high-frequency operation.

## Introduction

Since Landauer’s work in the 1950s^1^, we know that electric transport can be described by the scattering of electronic waves, in close analogy with the transmission of light in matter. The electron–photon analogy is even more adequate in graphene due to the linear dispersion of massless Dirac fermions and their sublattice pseudospin polarization^2^. For example, the refraction of Dirac fermion at a p–n junction obeys electronic variants of Snell–Descartes and Fresnel relations^3–6^ with an optical index proportional to the Fermi energy. Negative refraction and strong forward focusing effects could be measured^7,8^ using high-mobility hBN-encapsulated graphene^9,10^. Dirac fermion optics (DFO) naturally explains the large transmission of a Klein-tunneling potential barrier^11–18^, when regarded as an optical plate. The next step is to adapt the optics toolbox to Dirac fermions using p–n junctions as high-transmission diopters and electrostatically-shaped Klein tunneling barriers to implement basic refracting functions like reflectors.

Ballistic transistors exploiting total internal reflection across a prism-like, saw-tooth-shape, barrier have been proposed^19,20^ and demonstrated^21^ showing however rather limited on/off capabilities. Similar geometries, combining p–n junctions and graphene-edge reflections, have been proposed^22–24^ and measured^25–27^. They eventually suffer from spurious edge scattering^28,29^. Very recently, three-terminal ballistic switches have been demonstrated^29,30^, showing robust on/off ratios ~5. More involved DFO-based systems have also been considered, like Mie-scattering devices^31^, pinhole collimators^32,33^, Dirac fermion microscopes^34^, and chaotic DFO systems^35^. Beyond graphene, the interest in DFO extends to black phosphorus^36^, borophene^37^, surface states of topological insulators^38^, or massive states of topological matter^39,40^.

Here, we put DFO principles to quantitative test in the stringent geometry of a corner reflector using state-of-the-art bottom-gated hBN-encapsulated graphene. Right-angle prism CRs of ref. ^20^ are two-dimensional (2D) Dirac fermion variants of lunar laser retro reflectors^41^, or radar corner reflectors^42^. When compared to their optic counterpart, graphene CRs benefit from a gate-tunable refraction index ratio, in a range *n*_r_ = −6 → 6 exceeding that of light refractors in the visible (*n*_*r*_ ≲ 2.3 in diamond) or infrared (*n*_*r*_ ≲ 4 in semiconductors) range. Full suppression of electronic transmission relies on multiple ballistic total internal reflections predicted for *n*_*r*_ ≳ 2.5 according to ref. ^20^. Here, we show that the residual CR transmission is ultimately limited by minute acoustic phonon scattering in the barrier, which acts as a cutoff time/length for these multiple internal reflections. This effect explains the modest on/off ratios of CR-transistors, which precludes their use for logic applications. When put in perspective with their excellent dynamical properties, it also promotes CRs as valuable ballistic phonon detectors at low temperature.

## Results

### Working principle

**Fig. 1:**
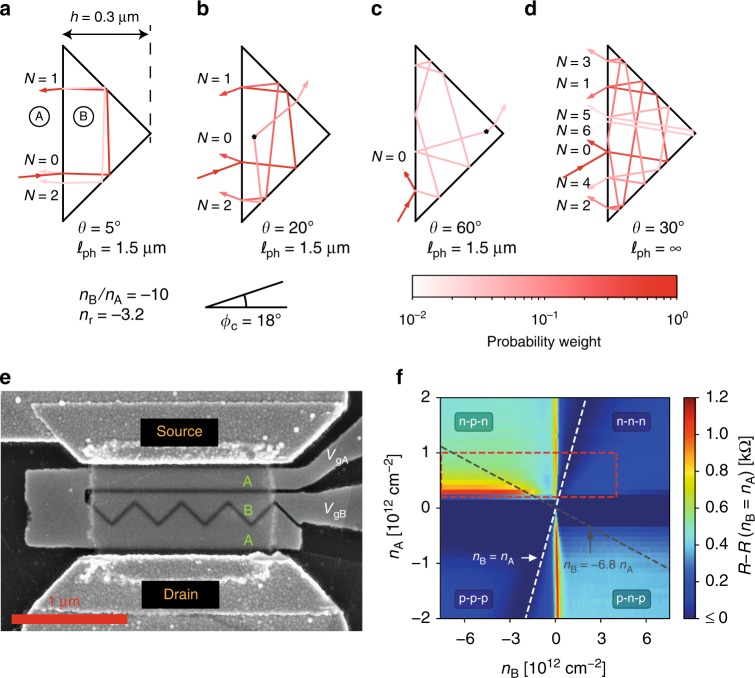
The corner reflector. **a**–**d** Calculated trajectories in a corner reflector with a critical angle *ϕ*_c_ = 18°, corresponding to a doping ratio of 10 between the regions *A* and *B*. At low incidence angle, the probability of the fermion to be reflected after one round trip is close to unity. When the incidence angle is slightly oblique, fermions have a high probability of staying trapped within the prism for multiple round-trips, and become susceptible to phonon scattering, which helps them escape the prism. At high incidence angle, the probability of being reflected before entering the prism is close to unity. **e** Annotated SEM micrograph of the device (sample CR-H9.4). Two bottom gates *V*_gA_ and *V*_gB_ allow for local control of the DFO refractive index in the access (*A*) and barrier (*B*) regions. hBN-encapsulated graphene is visible as a transparent rectangle, contacted at the edge by source and drain electrodes. **f** Color plot of the device resistance as a function of the charge carrier densities in regions *A* and *B*. The shading of the four quadrants is typical for a double-junction: low resistance (dark blue) in the unipolar quadrants and higher resistance (light blue) in the bipolar quadrants. The resistance minimum *R*(*n*_A_ = *n*_B_) (contact resistance) was subtracted from the data. The white dashed line indicates *n*_A_ = *n*_B_, consistent with the resistance minimum, whereas the black dashed line indicates *n*_B_ = −6*n*_A_, the criterion for total internal reflection. In the following we will focus on the data within the red dashed box

As an introduction to reflector principles, Fig. 1a–d shows calculated electron trajectories (Supplementary Note II and below) for typical incidence angles *θ* in the presence of a finite phonon scattering length $\ell _{{\mathrm{ph}}} = 1.5\,\mu {\mathrm{m}}$ (see below). At low incidence (*θ* = 5° in Fig. 1a) quasi-total reflection is achieved upon a single dwell cycle. The dwell length *L*_1_ = 2*h*, where *h* = 0.3 μm is the prism height, being smaller than $\ell _{{\mathrm{ph}}}$, reflection is insensitive to phonon scattering. At larger incidence (*θ* = 30° in Fig. 1d) a similar reflection amplitude requires *N* ~ 5 ballistic cycles, each cycle contributing to the total reflection as shown by the color code of rays in the figure. The *N*-path cycle length, *L*_*N*_ ≃ *NL*_1_ in right angle CRs, is independent of the impinging position and eventually exceeds $\ell _{{\mathrm{ph}}}$ leading to the breaking of ballistic trajectories. The two central sketches, for *θ* = 20° in Fig. 1b and *θ* = 60° in Fig. 1c, illustrate this effect. Scattering induces a finite barrier leakage after a cycle number *N* ~ $\ell$_ph_/2*h* ≃ 2.5. As a matter of fact, scattering breaks the regularity of ballistic trajectories eventually leading electron rays to enter the otherwise forbidden p^+^–n junction transmission window, for an incidence angle to the exit junction *ϕ* ≲ *ϕ*_c_ ≃ 18°. Scattering can be due to interface roughness, frozen disorder or, as in our experiment, to thermal phonons in which case a statistical averaging of scattering events becomes relevant. Note that due to pseudo-spin conservation, scattering in graphene has a drastic effect on electron trajectories, with a prominently ±*π*/2 momentum rotation^43^.

### Sample description

Figure 1e is a scanning electron microscope (SEM) image of sample CR-H9.4. The fabrication is detailed in the “Methods” section. Eight similar devices have been fabricated and characterized, which are described in Supplementary Note III. All samples are embedded in a three-port coplanar waveguide (see Methods) for DC, quasi-DC (10 kHz Lock-In measurements), and radio frequency (RF) characterization which are performed in a variable temperature 40 GHz probe-station. One distinguishes in Fig. 1e the semi-transparent hBN-encapsulated graphene sample of dimensions *L* × *W* = 0.8 × 1.6 μm. It has a bottom hBN thickness of 9 nm giving a gate capacitance *C*_*g*_ ≈ 3 mF m^−2^. The doping window, |*n*_A,B_| = 0.25 → 8 × 10^12^ cm^−2^, is limited by device integrity at high density and access resistance or spurious mirage effects due to charge puddles at low density. It corresponds to electronic wave lengths $\lambda _{{\mathrm{A}},{\mathrm{B}}} = \sqrt {4\pi /\left| {n_{{\mathrm{A}},{\mathrm{B}}}} \right|} = 15 \to 70\,{\mathrm{nm}}$, a widely tunable index ratio $n_{\mathrm{r}} = \sqrt {|n_{\mathrm{B}}/n_{\mathrm{A}}|} = 1 \to 6$, and n–p^+^ junction critical angles ${\mathrm{arcsin}}\,n_{\mathrm{r}}^{ - 1} = 10^ \circ \to 90^ \circ$. Also seen in Fig. 1e are the source and drain Cr/Au edge contacts, the refracting barrier gate (B) and the common source and drain access gate (A). Gate metallizations, of nominal thickness 25 ± 5 nm, are made of tungsten for low-frequency devices and gold for GHz devices. A thin slit of width 20 nm is etched to secure inter-gate isolation and achieve steep p–n junctions. Together with the small bottom-hBN thickness it defines the junction length *d* ≈ 30 nm (see a COMSOL electrostatic simulation and a discussion on screening in Supplementary Note IV) and provides high-transparency junctions of transmission ${\cal{T}}_{{\mathrm{np}}^ + }\sim 0.5$. Our CR design fulfills geometrical optics conditions, $d \lesssim \lambda _{{\mathrm{A}},{\mathrm{B}}} \ll h < \ell _{{\mathrm{ph}}}$, where $\ell _{{\mathrm{ph}}}(T) = 0.8 \to 24\,\mu {\mathrm{m}}$ in our working range *T* = 10 → 300 K^44^. The barrier consists of four connected right-angle prisms. Their overlap secures a minimum gate length of 100 nm preventing direct source-drain tunneling. The short access length of ~0.2 μm, favors ballistic transport with a cos *θ* distribution of the incidence angles. Owing to the n-doping of Cr/Au contacts, we work in the n–p^+^–n regime where the highly doped barrier fully controls the transmission ${\cal{T}}_{{\mathrm{CR}}}(n_{\mathrm{A}},n_{\mathrm{B}})$ of the *M*_A_ = *k*_A_*W*/*π* access modes (*M*_A_ = 40 → 80 for *n*_A_ = 0.25 → 1 × 10^12^ cm^−2^).

Figure 1f is a color plot of the quasi-DC CR resistance in a broad range of access and barrier doping. The vertical Dirac-peak resistance line at *V*_gB_ = 0 shows the independence of access and barrier doping control. The resistance vanishes at *n*_A_ = *n*_B_. We have subtracted a contact resistance *R*_c_ ~ 5 kΩ that can be minimized down to ~200 Ω using a better technology^30^ or sample design^44–46^. The CR effect shows up as a resistance resurgence for |*n*_B_| ≳ 6|*n*_A_|. Clearly visible in the n–p^+^–n regime, it is elusive in the p–n^+^–p regime, presumably due to spurious Klein-tunneling reflections at the n-doped contact. The red-dashed-rectangle in Fig. 1f delimits the working window analyzed below.

### The corner reflector transmission plateaus

**Fig. 2:**
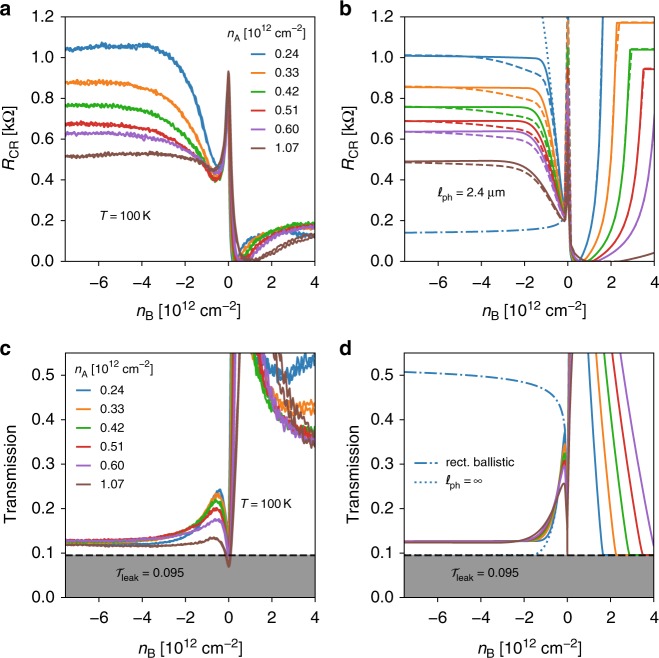
Corner-reflector transfer characteristics in the incoherent regime (*T* = 100 K): theory and experiment. **a**, **c** Experimental device resistance (quasi-DC) and transmission as a function of barrier doping *n*_B_ for various access doping *n*_A_ (c.f. red box in Fig. 1f). **b**, **d** Solid lines: resistance and transmission from simplified equation (Supplementary Equation-1), using a scattering length of $\ell$_ph_ = 2.4 μm and a junction length of *d* = 30 nm, saturating in the n–p^+^–n regime to the plateaus given by Eq. (1). Dashed lines: resistance from complete ray-tracing simulation. Dashed-dotted blue lines: resistance/transmission of a rectangular ballistic n–p–n barrier of same area and junction length and *n*_A_ = 0.24 × 10^12^ cm^−2^. Dotted blue lines: CR resistance/transmission with infinite scattering length in the same conditions. A leak transmission of 9.5% was taken into account in all simulations

Figure 2a shows a typical set of CR transfer characteristics *R*_CR_(*n*_A_, *n*_B_), taken at an intermediate temperature *T* = 100 K where *l*_ph_ ~ 2 μm. Different curves correspond to different access doping *n*_A_ > 0. The Dirac peak at barrier charge neutrality is accompanied by resistance resurgences for |*n*_B_| > *n*_A_ which are signatures of the CR effect. CR is fully developed in the bipolar regime, with resistance plateaus for *n*_B_ ≲ −6*n*_A_ ≃ −3 × 10^−12^ cm^−2^. The plateau resistance decreases for increasing access doping in accordance with the refraction principles prescribing a large index contrast. It eventually overshoots the Dirac point resistance, illustrating the efficiency of CRs in controlling the barrier transmission. Taking the *n*_B_ = ±4 × 10^12^ cm^−2^ values as reference “on” and “off” states, we estimate an on-off ratio ~5 for the barrier resistance alone. The *n*_A_-dependence is mostly determined by the number *M*_A_ of access modes as seen in Fig. 2c which shows a doping-independent transmission plateau, *T*_CR_ = *R*_L_/(*R*_CR_ + *R*_L_) where *R*_L_ = *h*/4*M*_A_*e*^2^ is the Landauer resistance. Note that these resistance plateaus, which are the signature of CRs, were not reached in the early investigation of ref. ^21^ where $\sqrt {|n_{\mathrm{B}}/n_{\mathrm{A}}|} \lesssim 1.7$ remained below the CR regime conditions $\sqrt {|n_{\mathrm{B}}/n_{\mathrm{A}}|} \gtrsim 2.5$.

### Phonon scattering limited corner reflector transmission

**Fig. 3:**
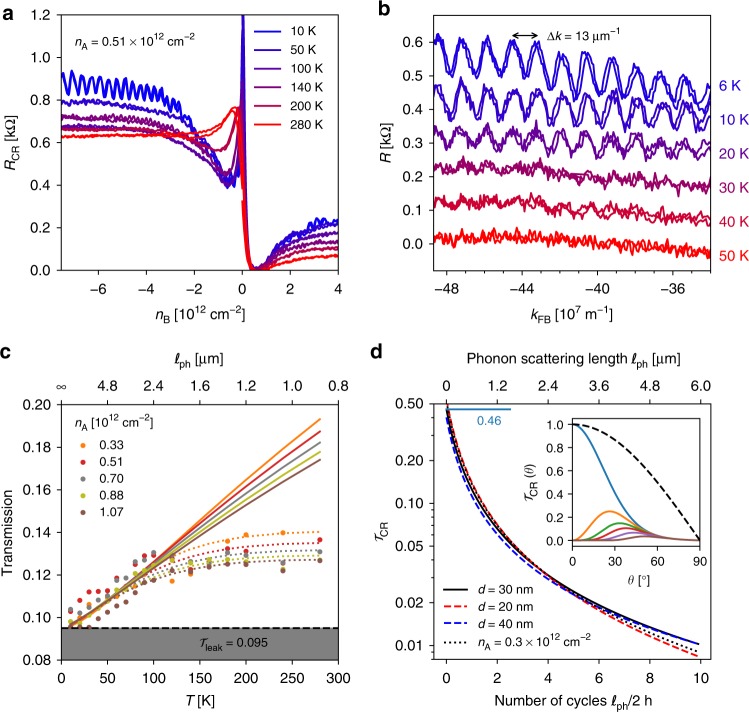
Temperature dependence of the corner-reflector transmission: coherence, phonon scattering and saturation. **a** Device resistance (quasi-DC) at various temperatures *T* = 10…280 K. The plateau resistance decreases as the temperature increases. **b** At low temperatures, Fabry–Pérot type oscillations (Δ*k* = 1.3 × 10^7^ m^−1^) are observed in the resistance, they disappear at *T* ≈ 40 K. (Resistance offset for clarity.) **c** Plateau transmission as a function of temperature for various access doping values *n*_A_. Solid lines and dotted lines are calculated using Eq. (1) with *d* = 30 nm and *d* = 30 nm + 2.25 pm/K^2^ × *T*^2^ respectively. **d** Plateau transmission Eq. (1) as a function of the number of round-trips in the CR, compared to the transmission of a single p–n junction ${\cal{T}}_{{\mathrm{np}}^ + } = 0.46$ (light blue bar). We used a junction length of *d* = 30 nm and doping values *n*_A_ = 0.5 and *n*_B_ = −5 × 10^12^ cm^−2^ (black line). Varying the junction length (red and blue dashed lines) or the doping (black dotted line) only has a small effect on the overall shape of this curve. Inset: CR transmission as a function of incident angle, for various numbers of cycles. Blue line: transmission of a single n–p junction (zero cycles). Other color lines represent how the transmission is increasingly suppressed when increasing the number of round-trips (1, 2, 3, 5, 10). The transmission is weighted in Eq. (1) by the cos *θ* factor (black dashed line)

The CR transmission can be calculated with semi-classical ray-tracing simulations^20,29^, taking into account the number of ballistic modes in the access region, the transmission ${\cal{T}}(\phi )$ of the three junctions as a function of refraction angle *ϕ* and multiple reflections inside the barrier. Accounting for residual quasi-elastic phonon scattering in this model amounts to randomizing the direction of propagation of the fermions after traveling an average length $\ell _{{\mathrm{ph}}}$ (details in Supplementary Note II). In the special case of a right-angle prism, due to the existence of recurrent trajectories, an analytic formula for the prism transmission could be derived (see Supplementary Equation-1), which reduces to the following compact formula in the CR regime:

1 $$\begin{array}{*{20}{c}} {{\cal{T}}_{{\mathrm{CR}}}(T) \approx {\int}_{\mathrm{0}}^{\frac{\pi }{2}} {T_{{\mathrm{np}}}} (\theta )\left[ {1 - {\cal{T}}_{{\mathrm{np}}}(\theta )} \right]^{\ell _{{\mathrm{ph}}}/2h}{\mathrm{cos}}\,\theta \,d\theta } & {\left( {{\mathrm{for}}\;|n_{\mathrm{B}}| \gtrsim 6|n_{\mathrm{A}}|} \right),} \end{array}$$

where the cos*θ* factor accounts for a uniform distribution of the transverse momentum (*k*_*y*_ = *k*_A_ sin*θ*) in the access. A marked difference between Supplementary Equation-1 and Eq. (1) is that the Snell–Descartes relation (*k*_A_ sin *θ* = *k*_B_ sin *ϕ*) no longer appears explicitly in the expression for the CR transmission, which becomes entirely determined by the junction transparency and mean-free path. Equation (1) accounts for the statistical nature of phonon scattering by interpolating the integer number of cycles *N* to continuous values $\ell _{{\mathrm{ph}}}/2h$. The $\ell _{{\mathrm{ph}}} = 0$ limit reduces to the bare n–p^+^-junction transparency $\langle {\cal{T}}_{{\mathrm{np}}}\rangle _\theta$. Following ref. ^20^, we use the Cayssol–Huard expression for the junction transmission^6^ (electronic variant of the Fresnel-relations in optics):

2 $${\cal{T}}_{{\mathrm{np}}}(\theta ) = 1 - \frac{{{\mathrm{sinh}}(\pi w\kappa ^{ + - }){\mathrm{sinh}}(\pi w\kappa ^{ - + })}}{{{\mathrm{sinh}}(\pi w\kappa ^{ + + }){\mathrm{sinh}}(\pi w\kappa ^{ - - })}},$$

with *κ*^*ρσ*^ = *k*_*p*_(1 + *ρ*cos*θ*) − *k*_*n*_(1 − *σ*cos*ϕ*) and *w* = *d*/2ln(10), where *d* is the junction length^20^. The link between the incident angle *θ* and the transmitted angle *ϕ* is given by the equivalent of the Snell–Descartes law: *k*_*p*_ sin *ϕ* = *k*_*n*_ sin *θ*. Equation (2) is suited for the asymmetric sharp junctions considered here. Alternatively, one can rely on the Cheianov–Fal’ko’s Gaussian expression for smooth symmetric junctions $\cal{T}$_np_(*θ*) ≃ exp(−*πk*_n_*d* sin^2^ *θ*)^5^, suitably modified for asymmetric doping^23,29^. As shown in the inset of Fig. 3d, ${\cal{T}}_{{\mathrm{np}}^ + }(\theta )$ vanishes for *θ* ≳ 50° in our sample. Equation (1) is very useful for the design of Dirac fermion reflectors and the analysis of their properties. As a practical criterion we can estimate the number of cycles, $N(\theta ) \simeq \left( {{\mathrm{log}}\epsilon - {\mathrm{log}}{\cal{T}}_{{\mathrm{np}}^ + }(\theta )} \right)/\left( {1 - {\cal{T}}_{{\mathrm{np}}^ + }(\theta )} \right)\sim 5$, needed to achieve a $1 - \epsilon = 0.99$ reflection coefficient for ${\cal{T}}_{{\mathrm{np}}^ + }(\theta )\sim 0.5$ (*θ* ≃ 30° in this example).

Figure 2b shows the calculated CR resistance *R*_CR_(*n*_A_, *n*_B_) for *d* = 30 nm, *h* = 0.3 μm, and $\ell _{{\mathrm{ph}}} = 2.4\,\mu {\mathrm{m}}$ mimicking *T* = 100 K conditions. A constant leakage transmission $\cal{T}$_leak_ = 0.095 (measured close to *T* = 0) has been added to account for device imperfections. The analytic formula (Supplementary Equation-1) quantitatively reproduces the main features of experimental data in Fig. 2a, including the plateau at negative *n*_B_. The plateau is a mere consequence of the saturation of ${\cal{T}}_{{\mathrm{np}}^ + }(\theta )$ in increasing barrier doping |*n*_B_| ≫ |*n*_A_| according to Eq. (2). In Supplementary Equation-1 the model also predicts a strong resistance resurgence in the n–n^+^–n regime which is not observed experimentally, a discrepancy that remains to be clarified. Such a contrast between unipolar and bipolar regimes may stress the importance of the negative barrier index, or equivalently pseudo-spin conservation, in enforcing CR reflection conditions. The ray-tracing simulations are added as dashed lines in the figure. The overlap of the two types of simulations confirms the relevance of Supplementary Equation-1 and Eq. (1) in describing right-angle prism CRs. Figure 2d shows that the calculated transmission ${\cal{T}}_{{\mathrm{CR}}}(n_{\mathrm{A}},n_{\mathrm{B}})$ is indeed independent of doping in the CR regime in agreement with experiment.

Also shown in Fig. 2b, d are the resistance/transmission of a conventional rectangular Klein-tunneling (KT) barrier (blue dashed-dotted lines) for *n*_A_ = 0.24 × 10^12^ cm^−2^. It is essentially controlled by the transmission of the n–p^+^ barrier-entry junction, the strong forward focusing entailing a quasi-total transmission at the parallel barrier-exit junction. Inspection highlights the strong suppression of transmission in CRs, well below the KT-barrier transmission value ${\cal{T}}_{{\mathrm{KT}}} \simeq 0.4$. It also shows that the resistance dips at *n*_B_ ≃ −*n*_A_ closely approach the rectangular barrier limit and can therefore be used to estimate the n–p^+^-junction transparency $\langle {{\cal{T}}_{{\mathrm{np}}^ + }} \rangle _\theta$. The fully ballistic case of ref. ^20^, ${\cal{R}}_{{\mathrm{CR}}}( {\ell _{{\mathrm{ph}}} = \infty })$ and ${\cal{T}}_{{\mathrm{CR}}}( {\ell _{{\mathrm{ph}}} = \infty })$ (blue dotted lines), is added for comparison. The parameter-free agreement between model and experiment concerns the existence of plateaus, their barrier doping threshold, as well as the access doping dependence. It constitutes a strong evidence that DFO principles can be implemented in a functional device on a quantitative basis. The value of the plateau transmission itself involves an extrinsic leakage ${\cal{T}}_{{\mathrm{leak}}}$, which sets a limit to the on/off ratio of CRs at low temperatures. It can be minimized with a better sample design minimizing edge effects, with e.g., a larger number of prisms (and extension *W*) or an edge-free Corbino geometry.

### Temperature dependence of corner reflector transmission

We now consider the effect of thermal electron and phonon excitations on the coherent and incoherent response in the CR transmission regime. Figure 3a is a plot of *R*_CR_(*n*_B_, *T*) at *n*_A_ = 0.5 × 10^12^ cm^−2^ in increasing temperatures. CR plateaus decrease with temperature implying a remarkable negative temperature coefficient of the resistance. At the lowest temperatures (*T* = 10 K in the figure), Fabry–Pérot oscillations superimpose on the plateaus, indicating constructive and destructive electronic interference. This distinctive property of CRs is elusive in obtuse-prism geometries^21^, it reflects the constant single-path dwell length *L*_1_ = 2*h* of right angle prisms (Fig. 1a). FP oscillations, mostly visible in the n–p^+^–n regime, are $k_{{\mathrm{p}}^ + }$ periodic in Fig. 3b with a period Δ*k* = 13 μm^−1^. Being independent of the access doping these oscillations can be unambiguously attributed to internal barrier reflections. They correspond to an optical length 2*π*/Δ*k* ≃ 0.5 μm approaching the geometrical single cycle length *L*_1_ = 2*h* = 0.6 μm. They vanish above 30 K consistently with the expected thermal smearing temperature *T*_coh_ = *π*ℏ*v*_F_/(*k*_B_*L*_1_) ≃ 40 K. The shape of the high-temperature characteristics, *T* ≳ 100 K in Fig. 3a, is qualitatively different: the Dirac peak and resistance dip are smeared and the CR merely behaves as a switch with a temperature-independent on/off ratio ≃5, reminiscent of that reported in the three-terminal ballistic switch of ref. ^30^.

Figure 3c gathers the measured plateau transmissions ${\cal{T}}_{{\mathrm{CR}}}(n_{\mathrm{A}},T)$ (for *n*_B_ < −5 × 10^12^ cm^−2^) over the full investigated access doping and temperature ranges. Solid lines are parameter-free theoretical predictions from Eq. (1) with *d* = 30 nm and $\ell _{{\mathrm{ph}}}(T) = 300/T \times 0.8\,\mu {\mathrm{m}}$ taken from ref. ^44^ measured in similar encapsulated samples. For simplicity we have neglected here deviations from the linear temperature dependence of the phonon scattering rates below the Bloch-Grüneisen temperature^47,48^. The good agreement of the *T*-dependence with our parameter-free model below 100 K supports the relevance of Eq. (1). Besides, we observe a saturation of transmission above 100 K at ${\cal{T}}_{{\mathrm{CR}}}^{sat} \simeq 0.035$ for $\ell _{{\mathrm{ph}}}(T)/2h \lesssim 4$, i.e., well above the model application range $\ell _{{\mathrm{ph}}}(T)/2h \gtrsim 1$. Such a transmission saturation cannot be explained by a thermal activation process which would involve an excess transmission rather than a deficit. As a hint, we observe a concomitant thermal smearing of the *n*_B_ = −*n*_A_ resistance dip for *T* > 100 K in Fig. 3a, which implies a decrease of the n–p^+^-junction transparency and an apparent thermal dilatation of the junction length *d*(*T*). In this interpretation the saturation of transmission results from a compensation of the phonon-induced barrier transparency by a temperature-enhanced junction opacity. As an illustration, we can reproduce in Fig. 3c (dotted lines), experimental data with Eqs. (1) and (2) taking an effective junction length d^*^ = 30 nm + 2.25 pm/K^2^ × *T*^2^. In this analysis, the room-temperature effective junction length (d^*^ ~ 200 nm at *T* = 280 K) would eventually approach the length *h* of the barrier itself which is qualitatively consistent with the observed smearing of Dirac point in Fig. 3a.

A proper account of the high-temperature data is beyond the validity domain of Eqs. (1) and (2), and the main scope of our paper which mostly focuses on phonon-scattering effects. Still we can speculate on possible physical explanations of the transmission saturation. One of them is electron–electron interactions, which may contribute in various aspects: an additional scattering in the barrier, hydrodynamic viscosity effects, or a modification of the refraction law at the p–n junctions. Electronic viscosity is reported in hydrodynamic experiments^49,50^, where shear effects are enhanced by inducing inhomogeneous flows downstream of a small current injector^49^ or across a constriction^50^. Gate-defined p–n junctions are not rigid boundaries and do not *per se* enforce inhomogeneous flows; flow inhomogeneity may result from interactions, in which case viscosity appears as a second-order correction in electron–electron interactions. Electron–electron scattering in the barrier occurs over a length $\ell _{{\mathrm{e}} - {\mathrm{e}}}\sim \hbar v_{\mathrm{F}}E_{\mathrm{F}}/(\alpha k_{\mathrm{B}}T)^2$^49,51^ with $\alpha = e^2/(4\pi \epsilon _{{\mathrm{BN}}}\hbar v_{\mathrm{F}}) \simeq 0.68$ (for $\epsilon _{{\mathrm{BN}}} \simeq 3.2\epsilon _0$). Considering the large doping of the CR barrier (*E*_F_ ≳ 280 meV for *n*_B_ ≳ 6 × 10^12^ cm^−2^), the direct e-e scattering effect is negligible at low temperature with $\ell _{{\mathrm{e}} - {\mathrm{e}}} \gtrsim 2\,\mu {\mathrm{m}} > \ell _{{\mathrm{ph}}}$ below 100 K. If e-e collisions are as efficient as e-ph collisions in defocussing electron beams in the barrier, they should contribute as an additional leakage at high temperature, which is opposite to the experimental trend of a saturation. The same argument holds for optical phonon scattering which is expected to contribute mainly at room temperature^52^. Now we cannot exclude an additional effect of interactions in renormalizing the p–n junction transmission itself beyond the non-interacting picture governed by momentum and pseudo-spin conservation. To the best of our knowledge, this effect has not been considered theoretically. Here we fit finite-temperature data by introducing an empirical *T*^2^ correction in the effective junction length d^*^ entering the non-interacting Eq. (2). Such a *T*^2^ dependence may be regarded as a hint of an interaction effect. Angle-resolved transmission measurements are needed to address this issue.

The good overall agreement between experiment and scattering theory in Figs. 2 and 3 consolidates the relevance of Eqs. (1) and (2) in describing refraction properties of Dirac fermions at low temperature. In particular it rules out the importance of junction roughness (see ref. ^53^) or frozen disorder in controlling CR’s transmission. The former should lead to appreciable deviations from Fresnel relations and the latter to a saturation of the phonon mean-free-path effect at low temperature, both of which are not observed. The effect of interface disorder is indeed minimized here by a careful back-gate nano-patterning (Supplementary Note V) and its residual impact is reduced by a smooth junction effect with $d\sim \lambda _{{\mathrm{p}}^ + }$. For a more detailed discussion of non-idealities in DFO devices, we refer the reader to ref. ^29^. The effect of frozen disorder is minimized in our high-mobility encapsulated graphene samples, with a mobility requirement of *μ* ≳ 2 × 10^5^ cm^2^ V^−1^ s^−1^.

The DFO principles at work in CRs are summarized in Fig. 3d which shows the calculated plateau transmission ${\cal{T}}_{{\mathrm{CR}}}\left( {\ell _{{\mathrm{ph}}}/2h} \right)$ of Eq. (1) (solid black line). Additional lines have been drawn for nearby device parameters including the junction length; they illustrate the robustness of the CR effect, and its sensitivity as a ballistic-length meter to multi-cycle internal reflections. The inset shows ${\cal{T}}_{{\mathrm{CR}}}(N,\theta )$ in increasing internal cycle number *N* = 0–10. The *N* = 0 case is the bare n–p^+^ junction transmission collimating incident electrons within a ±50° aperture angle. It yields an angle-average transmission ${\cal{T}}_{{\mathrm{np}}^ + } \simeq 0.46$ (blue bar in the main panel). Already prominent at *N* = 1 for *θ* ≲ 10°, the suppression extends to a wider angular domain in increasing *N* reducing to a residual leakage ${\cal{T}}_{{\mathrm{CR}}} \lesssim 0.01$ at about *θ* ~ 45° for *N* = 10.

### Dynamical properties of corner reflectors

**Fig. 4:**
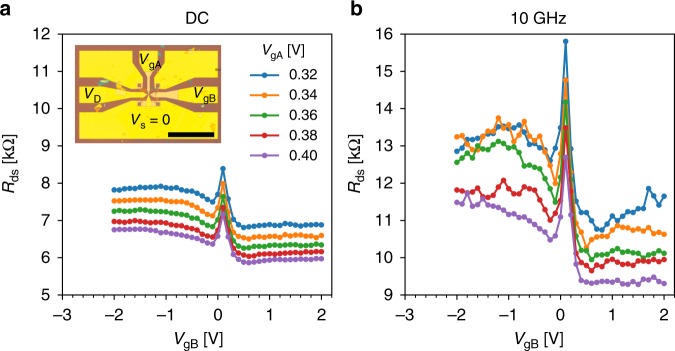
High-frequency corner reflectors. **a** DC source-drain resistance *R*_ds_ of sample CR-AuEG-17.ML (see Supplementary Note III) as a function of barrier gate voltage *V*_gB_ for various access gate voltages. Inset: Optical image of the sample embedded in the coplanar wave guide. Scale bar is 200 µm. **b** High frequency source-drain resistance

Owing to the large ratio of Fermi velocity *v*_F_ ≃ 10^6^ m/s and sound velocity *s* = 2 × 10^4^ m/s in graphene, corner reflectors are expected to have a picosecond time response, with a transit frequency $f_T \simeq v_{\mathrm{F}}/\ell _{{\mathrm{ph}}}\gtrsim 100\,{\mathrm{GHz}}$ and a phonon time-of-flight *τ* ≃ *h*/*s* ≃ 15 ps. In order to assess this high-speed property we have fabricated a few CRs equipped with low-resistance gold gates suitable for GHz operation (see Supplementary Note III). Figure 4 compares the DC and 10 GHz resistance *R*_ds_ at *T* = 60 K showing that CR properties are essentially preserved at 10 GHz. Note that finite frequency effects in the channel current or evanescent waves in the gate-charge distribution^54^ can be safely neglected in our submicron-size device. This observation is consistent with the above estimate and theoretical predictions in refs. ^55,56^; it shows that CRs are as fast as conventional graphene transistors (see e.g., refs. ^57,58^).

## Discussion

The combined high speed and phonon-scattering sensitivity of CRs offer promising perspectives in terms of ballistic phonon sensing. As a matter of fact, the phonon ballistic length, which is limited to a few hundreds of nanometers at room temperature, can exceed the CR barrier length at low temperature due to decreasing phonon-electron and phonon-phonon scattering rates^59^. Phonon time of flight measurements can thus be envisioned by merely monitoring transient CR resistance dips following the propagation of a remote phonon wave created by e.g., an optical pulse. One could consider phonon pulses of duration 50 ps, compatible with laser excitation, flying between two CRs separated by a distance of 1 μm. A transient temperature elevation of 10 K, or phonon population increase of $1/4\pi \times \left( {k_{\mathrm{B}}T/\hbar s} \right)^2{\int}_0^\infty {x/\left( {e^x - 1} \right)dx} \sim 5 \times 10^{10}\;{\mathrm{cm}}^{ - {\mathrm{2}}}$, would correspond to a transient phonon number Δ*N*_ph_ ~ 500 in the CR detector, to be detected by a (*v*_F_/*s* ≃ 50 times) larger number of electrons with a good signal to noise ratio. In this application one would benefit from the good sensitivity of CRs at low temperature and their low noise level as metallic detectors.

It is worthwhile comparing the corner reflector with a more standard ballistic device such as the switch of ref. ^30^. While the CR works with a strongly refracting barrier |*n*_B_| ≳ 6|*n*_A_|, the switch operates in the opposite limit $|n_{\mathrm{B}}| \lesssim |n_{\mathrm{A}}|$; CR enhances the effects of multiple internal reflections, the switch avoids them. This qualitative difference is illustrated in the barrier-gate-voltage dependence of the resistance: the corner reflector shows a resistance dip followed by a plateau in Fig. 2a, whereas the switch has a monotonic dependence (Figure 4 of ref. ^30^), which is reminiscent of that of two-terminal rectangular-gate Klein tunneling transistors.

To conclude, we have demonstrated Dirac fermion corner reflectors using state-of-the-art bottom-gate-defined high-mobility graphene nano-transistors. We have characterized the CR transmission over a broad range of access/barrier doping, temperatures, and frequencies. We have unveiled the existence of CR transmission plateaus, signature of total internal reflection in the strongly refracting regime (refraction index contrast window *n*_r_ = 2.5 → 6) and demonstrated the dependence of their amplitude on the phonon scattering length in quantitative agreement with a simple scattering theory. Our modeling is further supported by the observation of Fabry–Pérot oscillations in the quantum coherent regime which provides an independent measurement of the single-path dwell length that is consistent with the multi-path length measured by the phonon scattering effect. This full control of the geometrical and coherent optics regimes strengthens confidence in the DFO principles and opens the way to their harnessing for fundamental physics such as ballistic phonon transport. In addition we report on the saturation of CR transmission at high temperature attributed to a thermally induced junction opacity. Our experiment explains the longstanding issue of the finite on/off switching capabilities of reflectors which is limited at low temperature by device imperfections and at high temperature by the finite phonon scattering rate. We propose an application of reflectors as ballistic phonon sensors, relying on their high speed and phonon-scattering sensitivity below 100 K. CRs enrich the family of Klein-tunneling based devices, including the recently demonstrated ballistic switches^30^, contact barrier transistors^58^, and Zener–Klein transistors^45^. Besides, the quantitative approach of DFO demonstrated in this work motivates further basic-physics studies in other Dirac matter systems such as topological materials or eventually bilayer graphene where refraction laws are modified due to anti-Klein tunneling effects^3^.

## Methods

### Corner reflector fabrication techniques

For the fabrication of nano-structured bottom gate electrodes, thin (~30 nm) films of tungsten (W) or gold (Au) were deposited using sputtering or evaporation on a high-resistivity Si/SiO_2_ substrate. The patterning was done using two separate electron beam lithography (EBL) steps with positive poly(methyl methacrylate) (PMMA) resist for the 20 nm sawtooth line (defining the gap between the two gates) and the rough structures, each followed by reactive ion etching (SF_6_ plasma for W^58^, Ar plasma for Au). In order to contact the gate electrodes with our GHz probe tips, we deposited a thicker (150 nm) layer of Au all over the coplanar waveguide (CPW) leading to the sample center (see inset, Fig. 4a). Prior to subsequent fabrication steps, the gate electrodes were evaluated using SEM and tested for short circuits.

High-mobility hBN-encapsulated graphene was fabricated according to ref. ^9^ and subsequently transferred using PPC-scotch tape-PDMS stamps^60^ on W electrodes and PMMA-poly(vinyl alcohol) stamps^9^ on Au electrodes. The stacks were then etched through an EBL-defined PMMA mask into a nearly rectangular shape covering only the active area (see Fig. 1a) of the device using a CHF_3_/O_2_ plasma. Finally, Cr/Au (5/100 nm) drain and source contacts were deposited after the last EBL step.

### DC, quasi-DC, and RF characterization

The device characterization was carried out in a Janis cryogenic probe station operating between 6 and 300 K. The gate voltages were controlled using iTest Bilt voltage sources. The quasi-DC resistance of the device was measured using a Zurich Instruments HF2LI lock-in amplifier at 10.013 kHz with 5 mV amplitude in a voltage divider configuration.

For the quasi-DC experimental results shown in Figs. 1–3, the corner reflector resistance was defined as *R*_CR_(*n*_B_, *n*_A_) = *R*_raw_(*n*_B_, *n*_A_) − *R*_raw_(*n*_B_ = *n*_A_) for fixed values of *n*_A_, where *R*_raw_ is the measured (raw) device resistance. Here we assume that the central region of the device is fully transparent when the p–n junctions are removed by setting *n*_A_ = *n*_B_. This is why *R*_CR_(*n*_A_ = *n*_B_) = 0 in the figures. In the case of ideal, fully transparent contacts, the resistance of a ballistic two-terminal device is actually given by the Landauer resistance *R*_L_ (defined in the main text), which is why we add this value to *R*_CR_ when calculating the transmission of the corner reflector ${\cal{T}}_{{\mathrm{CR}}} = R_{\mathrm{L}}/(R_{{\mathrm{CR}}} + R_{\mathrm{L}})$.

The GHz frequency characterization was done using an Anritsu MS4644B vector network analyzer (VNA). In this case, the DC resistance was monitored simultaneously using another Bilt voltage source/meter, where the DC and GHz response of the device were decoupled from each other using bias tees. A standard short-open-load-reciprocal (SOLR) protocol was employed to calibrate the GHz wave propagation until the probe tips and the admittance parameters were de-embedded from parasitic capacitance by measuring a reference sample without graphene.

## Supplementary information

Supplementary Information

## Data Availability

The data that support the findings of this study are available from the corresponding author upon reasonable request.
