# Supplementary material for: A corner reflector of graphene Dirac fermions as a phonon-scattering sensor
Source: Nat Commun. 2019 Jun 3;10:2428. doi: 10.1038/s41467-019-10326-6 (PMC6547877; doi:10.1038/s41467-019-10326-6)
Supplement: Supplementary file 1 — Supplementary Information [file 41467_2019_10326_MOESM1_ESM.pdf]

A corner reflector of graphene Dirac fermions as a phonon-scattering sensor: Supplementary Material

H. Graef et al.

Supplementary Note I: Analytical modeling of Corner reflector transmission

A typical electron beam propagating in a right-angle corner reflector (CR) is depicted in Supplementary Fig.1. The trajectory involves a transmission through the straight junction (1), from the access (A) to the barrier (B), which collimates the incoming beam from θ to $\phi < \theta$, where $k_{\mathrm{A}} \sin\theta = k_{\mathrm{B}} \sin\phi$, and two subsequent reflections from the tilted junctions (2) and (3). If the incidence to the tilted junctions deviated form perpendicularity there is a finite reflection probability and if the deviation is greater than the critical angle ϕ_c the reflection probability reaches unity. In the 90° apex prism, it can be easily shown that the fermion is reflected back into the prism with its original angle ϕ (with a change of sign, which is insignificant due to the symmetry of the prism), i.e. the trajectory is *recurrent*.

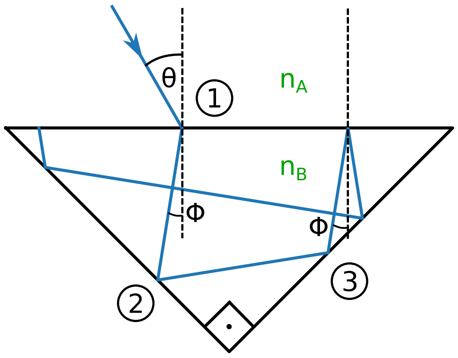

Supplementary Figure 1: CR trajectory with two round-trips in the 90° apex prism. Here $n_{\mathrm{B}} = -10\ n_{\mathrm{A}}$, $\phi_c = 18°, \theta = 30°$, $\phi = 9°$.

We define the transmission probabilities for the three junctions, which, for fixed n_{A} and n_{B}, only depend on the initial incidence angle θ: $\mathcal{T}_1(\theta)$, $\mathcal{T}_2(\theta)$, $\mathcal{T}_3(\theta)$. The incident angles on junctions (2) and (3) are simply given by $\phi_2 = 45° - \phi$ and $\phi_3 = 45° + \phi$. The probability for the fermion of being reflected back to the access after one round-trip is $\mathcal{R}_{\mathrm{cycle}} = (1 - \mathcal{T}_2)(1 - \mathcal{T}_3) \times \mathcal{T}_1$, where we dropped the θ-dependence for better readability. The probability to stay in the prism is $P_{\mathrm{stay}} = (1 - \mathcal{T}_2)(1 - \mathcal{T}_3)(1 - \mathcal{T}_1)$. For the total reflection of the prism, we therefore find:

$$\begin{aligned}\mathcal{R}_{\mathrm{tot}}(\theta) &= 1 - \mathcal{T}_1(\theta) + \mathcal{T}_1(\theta) \sum_{i=0}^{N-1} P_{\mathrm{stay}}^i(\theta)\mathcal{R}_{\mathrm{cycle}}(\theta) \\ &= 1 - \mathcal{T}_1(\theta) \left[1 - \mathcal{R}_{\mathrm{cycle}}(\theta)\frac{1 - P_{\mathrm{stay}}^N(\theta)}{1 - P_{\mathrm{stay}}(\theta)}\right] \quad ,\end{aligned}$$

where the first $1 - \mathcal{T}_1(\theta)$ accounts for the probability of reflection before entering the prism and N is the number of round-trips. We deliberately choose to sum over the cycle *reflections*, so that – if the sum is truncated at a given number N of round-trips – the remaining probability weight counts towards the *transmission*:

$$\mathcal{T}_{\mathrm{tot}}(\theta) = \mathcal{T}_1(\theta) \left[1 - \mathcal{R}_{\mathrm{cycle}}(\theta)\frac{1 - P_{\mathrm{stay}}^N(\theta)}{1 - P_{\mathrm{stay}}(\theta)}\right] \tag{1}$$

In the total internal reflection regime ($|n_{\mathrm{B}}| \geq 6\ |n_{\mathrm{A}}|$), where the impinging beam is sufficiently collimated by junction (1), one has $\mathcal{T}_2 = \mathcal{T}_3 = 0$, $\mathcal{R}_{\mathrm{cycle}} = \mathcal{T}_1$ and $P_{\mathrm{stay}} = 1 - \mathcal{T}_1$. Equation 1 then simplifies to:

$$\mathcal{T}_{\mathrm{CR}}(\theta) = \mathcal{T}_1(\theta) \left[1 - \mathcal{T}_1(\theta)\right]^N \tag{2}$$

It can be easily shown from basic geometry that trajectories with near-normal ϕ all have approximately the same length L_1, which means that the number of round-trips before a scattering event can be re-written as $N \sim l_{\mathrm{ph}}/L_1$. By substituting the discrete N to a continuous $\mathcal{N} = l_{\mathrm{ph}}/2h$ we account for the statistical averaging of time-dependent phonon scattering events :

$$\langle \mathcal{T}_{\mathrm{CR}} \rangle_\theta = \int_0^{\pi/2} \mathcal{T}_1(\theta) \left[1 - \mathcal{T}_1(\theta)\right]^{l_{\mathrm{ph}}/2h} \cos\theta \; d\theta \tag{3}$$

This equation is reproduced in the main text, where $\mathcal{T}_1(\theta)$ is replaced by $\mathcal{T}_{\mathrm{np}}(\theta)$

Supplementary Note II: Full ray-tracing simulations

A complete ray-tracing simulation was carried out for the data obtained at $T = 100$ K. The simulation takes into account a 90° apex-prism of height $h = 0.3$ μm and sums over all positions along the y-axis and over all incident angles in order to find the overall transmission of the device:

$$\langle \mathcal{T}(n_{\rm A}, n_{\rm B}) \rangle_\theta = \frac{1}{2h} \sum_0^h \Delta y \sum_{-\pi/2}^{\pi/2} \Delta\theta \;\; \mathcal{T}(n_{\rm A}, n_{\rm B}, y, \theta) \cos(\theta) \;, \tag{4}$$

where $\mathcal{T}(n_{\rm A}, n_{\rm B}, y, \theta)$ is the probability for the fermion to be transmitted across the prism for the trajectory defined by $n_{\rm A}, n_{\rm B}, y$ and θ. The trajectories are calculated using the Snell-Descartes law and the transmission probabilities for the three pn-junctions are given by the Cayssol-Huard formula (c.f. Supplementary Figure 2). We used discretizations of $\Delta y = 5$nm and $\Delta\theta = 0.001$.

For the ray-tracing results shown in Fig. 2b of the main text, phonon scattering was incorporated in the simplest possible way: the fermion is transmitted across the prism with a probability of unity after traveling the length $\ell_{\rm ph}$. We have carried out similar simulations using a Monte Carlo approach, sampling the scattering direction from a uniform angular distribution and sampling the dwell length from an exponential distribution. Eventually, these increasingly complicated models lead to a similar linear $\mathcal{T}_{\rm CR} \propto 1/\ell_{\rm ph}$ relation that is shown in fig. 3c of the main text, up to a scaling factor ~ 1 that compensates for the overestimation of the residual transmission by the simple approach.

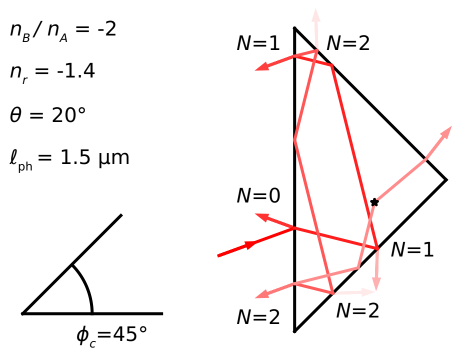

Supplementary Figure 2: An example ray-tracing simulation for $|n_{\rm B}/n_{\rm A}| = 2 < 6$. In this regime, total internal reflection on the right side of the prism is not guaranteed and fermions can be transmitted for certain incidence angles.

Supplementary Note III: Overview of the CR samples used in this work

The robustness of the results presented in the main text can be assessed in the light of the CR series investigated in this work. In total, eight samples were fabricated (see Supplementary Table I and Supplementary Fig.4), two of them did not have a working source/drain contact and one of them suffered from an inter-gate leak after the final fabrication step. Out of the remaining five, three showed the desired reflection effect, illustrated in Supplementary Fig.3, where we confirm that this effect is purely due to the sample geometry (a 90° prism in all shown cases), whereas the other two show a similar gate-voltage dependence, but slightly weakened, possibly due to insufficient mobility and/or geometrical imperfections, c.f. corresponding SEM pictures in Supplementary Fig.4.

sample	gate material	contact material	angle	bottom hBN thickness [nm]	n_A $[10^{12}\mathrm{cm}^{-2}]$	T [K]
CR H4 1x3	W	Cr/Au	90°	6	-0.17	20
CR H5 2x3	W	Cr/Au	90°	25	-0.11	20
CR H8.2	W	Ti/Al	90°	8	defective	
CR H8.4	W	Ti/Al	90°	∼12	defective	
**CR H9.4**	**W**	**Cr/Au**	90°	**9**	**0.33**	**60**
CR-AuEG-11.MC	Au	Cr/Au	120°	21	defective	
CR-AuEG-17.BL	Au	Cr/Au	90°	19	-0.34	7
**CR-AuEG-17.ML**	**Au**	**Cr/Au**	90°	**5**	**0.85**	**60**

Supplementary Table I: List of CRs fabricated in this study. n_A and T denote the charge carrier density (positive for electron, negative for hole doping) and the working temperatures used in Supplementary Figure 3. The 2 samples analyzed in the main text are highlighted in bold.

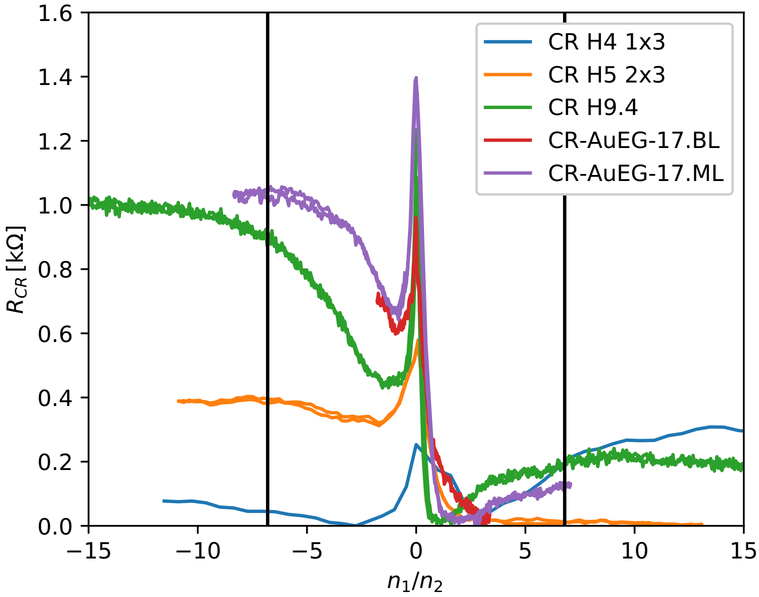

Supplementary Figure 3: Two terminal resistance of corner reflector samples at their working point (c.f. density n_A and temperature T in Supplementary Table I). For comparability, the minimum resistance was subtracted and the data is plotted as a function of the dimensionless density ratio $n_\mathrm{B}/n_\mathrm{A}$. The vertical black lines at $|n_\mathrm{B}/n_\mathrm{A}| = 6.8$ indicate the theoretical expectation for total reflection. We systematically observe a much stronger effect for negative $n_\mathrm{B}/n_\mathrm{A}$, i.e. for bi-polar junctions.

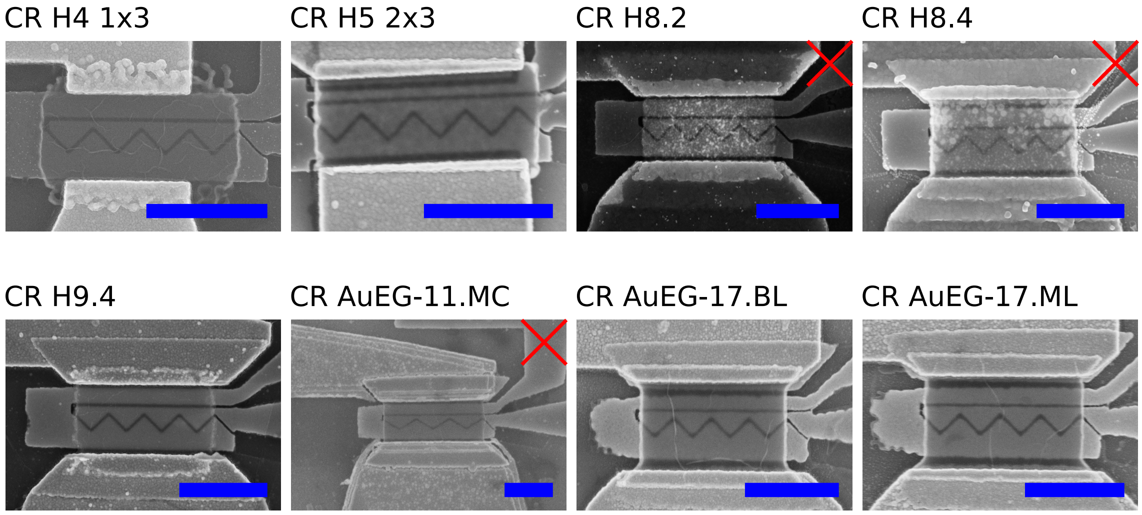

Supplementary Figure 4: SEM pictures of all fabricated devices. Blue scale bar corresponds to 1 μm, red crosses indicate defective samples.

Supplementary Note IV: Potential profile of the p-n junction

The effective length of the p-n junction can be estimated using a COMSOL finite element model, shown in Supplementary Figure 5a. Here the gap between the two bottom gates is denoted d_0 and the thickness of the hBN dielectric layer is t.

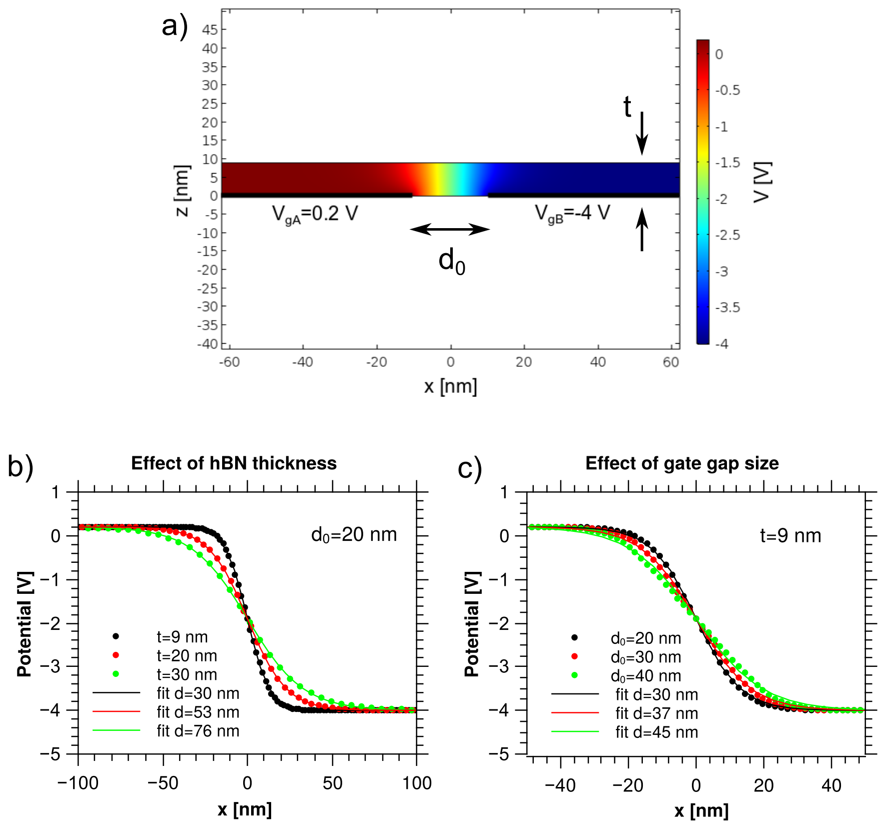

Supplementary Figure 5: Estimating the effective junction length. (a) Color plot of the electric potential for a gate separation of $d_0 = 20$ nm and a hBN thickness of $t = 9$ nm. (b) Potential on the hBN surface for various hBN thicknesses. (c) Potential on the hBN surface for various gate separations.

The potential profile in the graphene sheet is plotted in Supplementary Figures 5 for various values of d_0 and $t \lesssim d_0$. It is fitted using a Fermi type function:

$$V(x) = V_{\mathrm{gA}} + \frac{V_{\mathrm{gB}} - V_{\mathrm{gA}}}{1 + e^{-2\ln(10)x/d}} \tag{5}$$

where the pre-factor $2\ln(10)$ in the exponential ensures that d corresponds to the distance over which the potential reaches 90% of its final value. Comparing the panels (b) and (c) shows that the hBN thickness plays a crucial role for the sharpness of the p-n junction (more crucial than the gate separation). For the parameters of device CR-H9.4 ($d_0 = 20$ nm and $t = 9$ nm), we deduce an effective junction length of $d = 30$ nm.

The simulation does not take into account the screening due to the finite charge carrier density in the graphene sheet. It amounts to reducing the effective junction length below the above estimate. As shown in reference [1] finite screening length effects show up in the limit $d_0 \ll t$, resulting in an effective junction length $d \sim t$.

Supplementary Note V: Assessing junction roughness

While a recent publication [2] discusses the difficulty of creating straight, gate-induced p-n junctions defined by photolithography and using a thick SiO_2-layer as a gate dielectric, we overcome these limitations by means of electron beam lithography with nanometer resolution and by using only thin and flat as-exfoliated hBN crystals as gate dielectric. Supplementary Figure 6 shows a SEM picture of our gate electrodes taken before the deposit of the van der Waals heterostructure (left) and a zoom on one of the gaps between the access and the barrier gates (right). The separation between the two electrodes is $d_0 \approx 20$ nm. The p-n junction roughness should therefore be comparable to that achieved with cleaved-edge graphite gates in Ref. [2]. We note that in this work, the use of graphite gates was not an option, because they are not compatible with our high-frequency measurements.

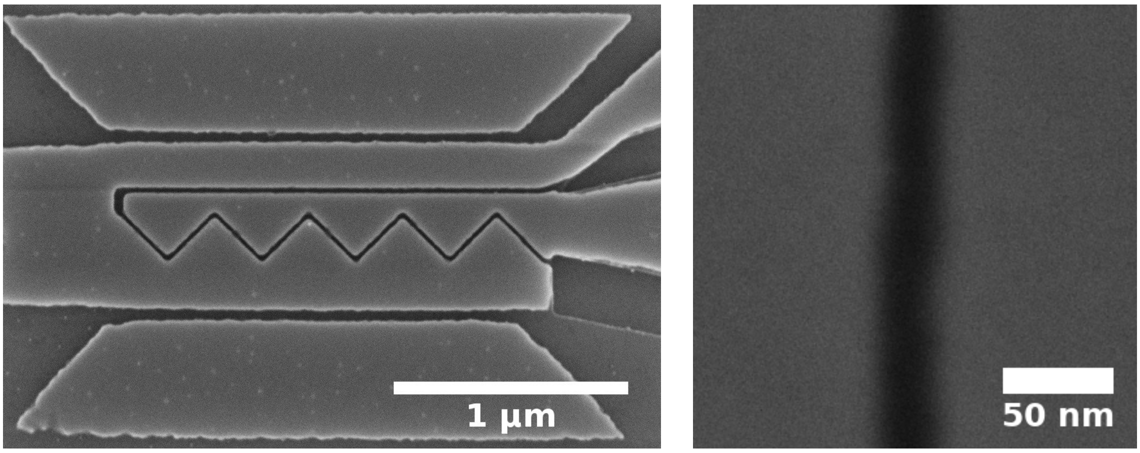

Supplementary Figure 6: **Left:** SEM picture of the gate electrodes of sample CR H9.4 before deposit of the hBN-graphene-hBN stack. The trapezoid shape pads on the top and the bottom only serve as mechanical support. **Right:** Zoom on the gap between the two gate electrodes.

Supplementary References

[1] Zhang, L.M., Fogler, M.M. Nonlinear screening and ballistic transport in a graphene p-n junction. *Phys. Rev. Lett.* **100**, 116804 (2008).

[2] Zhou, X. et al. Atomic Scale Characterization of Graphene p-n Junctions for Electron-Optical Applications. *ACS Nano* **13**, 2558-2566 (2019).
